# KGDCMI: A New Approach for Predicting circRNA–miRNA Interactions From Multi-Source Information Extraction and Deep Learning

**DOI:** 10.3389/fgene.2022.958096

**Published:** 2022-08-16

**Authors:** Xin-Fei Wang, Chang-Qing Yu, Li-Ping Li, Zhu-Hong You, Wen-Zhun Huang, Yue-Chao Li, Zhong-Hao Ren, Yong-Jian Guan

**Affiliations:** ^1^ School of Information Engineering, Xijing University, Xi’an, China; ^2^ College of Grassland and Environment Sciences, Xinjiang Agricultural University, Urumqi, China; ^3^ School of Computer Science, Northwestern Polytechnical University, Xi’an, China

**Keywords:** circRNA–miRNA interaction, circRNA, deep neural network, graph embedding, K-mer

## Abstract

Emerging evidence has revealed that circular RNA (circRNA) is widely distributed in mammalian cells and functions as microRNA (miRNA) sponges involved in transcriptional and posttranscriptional regulation of gene expression. Recognizing the circRNA–miRNA interaction provides a new perspective for the detection and treatment of human complex diseases. Compared with the traditional biological experimental methods used to predict the association of molecules, which are limited to the small-scale and are time-consuming and laborious, computing models can provide a basis for biological experiments at low cost. Considering that the proposed calculation model is limited, it is necessary to develop an effective computational method to predict the circRNA–miRNA interaction. This study thus proposed a novel computing method, named KGDCMI, to predict the interactions between circRNA and miRNA based on multi-source information extraction and fusion. The KGDCMI obtains RNA attribute information from sequence and similarity, capturing the behavior information in RNA association through a graph-embedding algorithm. Then, the obtained feature vector is extracted further by principal component analysis and sent to the deep neural network for information fusion and prediction. At last, KGDCMI obtains the prediction accuracy (area under the curve [AUC] = 89.30% and area under the precision–recall curve [AUPR] = 87.67%). Meanwhile, with the same dataset, KGDCMI is 2.37% and 3.08%, respectively, higher than the only existing model, and we conducted three groups of comparative experiments, obtaining the best classification strategy, feature extraction parameters, and dimensions. In addition, in the performed case study, 7 of the top 10 interaction pairs were confirmed in PubMed. These results suggest that KGDCMI is a feasible and useful method to predict the circRNA–miRNA interaction and can act as a reliable candidate for related RNA biological experiments.

## Introduction

Circular RNA (circRNA) is a kind of special single-stranded circular endogenous non-coding RNA molecule. Numerous studies have shown that circRNAs are highly conserved and biostable throughout the organism. CircRNAs were first found in RNA viruses in 1976. Sanger *et al.* (1976) thus described viroids as highly base-paired rod-like and single-stranded closed circular structures. In 1979, the study published by Hsu *et al.* provided electron microscopic evidence for the circular form of RNA (Hsu and Coca-Prados 1979). During the following 30 years, research on circRNAs has achieved some results, and a small number of circRNAs were discovered by accident (Nigro et al., 1991; Cocquerelle et al., 1992). However, during the early stages of research, circRNAs were considered to be abnormal splicing of genes. Although these early studies found and documented the existence of circRNAs and put forward guesses about their functions, the potential impact of circRNAs may have been largely ignored.

A surge in circRNAs research began around 2010 with the progress of RNA-seq technology and specialized computational pipelines, thus bringing circRNAs’ testing and sequence analysis back to biological research. CircRNAs’ importance gradually emerged, and many studies have indicated that circRNA has a higher stability structure than linear RNAs, which makes circRNA present the tissue-specific expression pattern and play a crucial role in some cell activities (Armakola et al., 2012; Li et al., 2015; Xu et al., 2015). In the meantime, the important biological functions of circRNA also connect it with the diagnosis and treatment of human diseases (Qu et al., 2015; Kulcheski et al., 2016). The circRNA–miRNA interaction is a classic and important aspect of circRNA-mediated gene regulation (Memczak et al., 2013). The circRNAs contain many miRNAs response elements making circRNA work as miRNA sponges (Hansen et al., 2013; Memczak, Jens, Elefsinioti, Torti, Krueger, Rybak, Maier, Mackowiak, Gregersen and Munschauer 2013). This leads to the circRNA binding to miRNA and repressing their function. MiRNA is one of the most important kinds of ncRNAs involved in several aspects of gene regulation in eukaryotes (Grishok et al., 2001; Siomi and Siomi 2010; Hayes et al., 2014). At present, there is numerous evidence showing that circRNAs are miRNA sponges resulting in the up-regulation of downstream proteins, which is closely associated with a variety of human diseases, such as type-2 diabetes, cardiovascular diseases, atherosclerotic vascular disease risk, and cancers (Zhang et al., 2018; Chen and Shan 2021). For example, circRNA can act as an oncogene in some tumors, causing the proliferation and metastasis of cancer cells. Yang *et al.* applied the RT-qPCR assay, CCK-8, wound-healing, and cell colony formation assay to detect the expression level and the effect of RNA molecular on cancer cell proliferation and metastasis, finally finding that circSPECC1 could promote the proliferation and migration of Bca and may be used as a new diagnostic biomarker and effective therapeutic target for some cancers (Yang et al., 2022). Chen *et al.* used quantitative real-time PCR and established the stable knockdown of circXRCC5 in U87 and U251 cells to assess the functions of RNA and detect the expression of circXRCC5 in glioma tissues, which proved that circXRCC5, as the sponge of miR-490-3p, regulates the expression of the downstream gene and promotes the progression of glioma (Chen et al., 2022). Tao *et al.* (2022) treated human lens epithelial cells with high glucose and detected gene expression by quantitative real-time polymerase chain reaction (PCR). The Cell Counting Kit-8 test, EdU test, and Western blot assay were used to detect cell proliferation and viability. The dual-luciferase reporter method and RNA immunoprecipitation assay were used to validate the target interactions. They finally clarified that circPAG1 can mediate the miR-211-5p/E2F3 axis to protect human lens epithelial cells from damage induced by high glucose (Tao et al., 2022).

The above studies indicate that the research of the circRNA–miRNA interaction can be a new biomarker for the treatment and diagnosis of diseases. However, the traditional biological experimental method is often expensive, time-consuming, and requires a lot of labor. Thus, predicting the interaction between miRNA and circRNA by computational methods is crucial for relevant research. At present, many prediction models have been applied in related fields. For example, Li *et al.* combined a variety of similarities and improved the traditional nonnegative matrix algorithm to predict disease-related miRNAs (Li et al., 2021). Ren et al. (2022) proposed a model named BioDKG-DDI, which combines multi-characteristic biochemical information and uses an attention machine to predict potential drug–drug interactions. Yin et al. (2020) proposed a computational method, NCPLP, which is based on the network consistency projection and label propagation to predict disease-associated microbes. Zhou et al. (2021) proposed a novel method that learns features through multiple kernel learning and deep autoencoder, finally predicting new microRNA -disease associations by reconstruction error. Pan et al. (2022) combined protein attribute and behavior vectors and used a deep neural network (DNN) to fuse the protein feature vector to predict the protein–protein interactions. Such computational models have achieved very successful results and provided an experimental basis for further studies.

Because the naming norms and studies on circRNA are not mature, the computational models regarding the circRNA–miRNA interaction are scarce compared with related fields. In recent years, as the amount of circRNAs data increased, a large number of databases have been developed to store information about circRNAs, like circR2Disease (Fan et al., 2018), circBase (Glažar et al., 2014), circRNA disease (Zhao et al., 2018), and Circbank (Liu et al., 2019). CircR2Disease is a manually curated database containing 661 circRNAs, 100 diseases, and 725 experimentally verified associations of circRNA-diseases. Each pair of associations in the circR2Disease database contains a brief description of the circRNA–disease relationship, gene symbol, expression patterns of circRNA, circRNA and disease name, experimental techniques, year of publication, and the PubMed ID. Circbase is an online database that includes thousands of circular RNAs in animals, which users can search the circRNA sequence, gene description, and circRNA ID. CircRNA disease is a database that documents a total of 354 circRNA–disease interaction pairs between 330 circRNAs and 48 diseases. Each pair includes the circRNA ID and circRNA name, the expression pattern and biological function of circRNA, disease name, and experimental detection techniques. Circbank is a comprehensive database with standard nomenclature, which includes approximately 140,000 human circRNAs, and the other five features of circRNAs. These databases are public and allow users to query the circRNA information based on different search criteria, which allows users to conveniently obtain data for research on circRNAs. This makes predicting the interaction of circRNA–miRNA by computational method possible.

For the purposes listed above, this study proposed a creative efficient computational method named KGDCMI to predict the circRNA–miRNA interactions based on multidimensional feature extraction and fusion. First, we use the K-mer algorithm to obtain the abundance of different fragment sequences in the whole RNA sequence, which represents the inherent attribute information of RNA molecules. To take full advantage of the biological information of circRNA and miRNA, we also constructed a Gaussian interaction profile kernel similarity matrix of each RNA. Second, the sparse autoencoder (SAE) was used to further extract features from redundant or sparse information to obtain the final attribute features. Next, we constructed a circRNA–miRNA bipartite graph to describe the associations between these molecules, in which each node represents an RNA molecule, and each link represents their interaction. Then we employed high-order proximity reserved embedding (HOPE), a graph-embedding algorithm to capture the description of behavior information between nodes from the interactions. At last, the DNN was used to objectively and automatically fuse multiple features and predict the potential circRNA–miRNA interactions effectively. To evaluate the performance of the proposed method comprehensively and fairly, the five-fold cross-validation was used in the experiment, and a variety of evaluation indicators were employed to evaluate the performance and practicability of the proposed method. As a result, an 89.30% area under the curve (AUC) and 87.67% area under the precision–recall curve (AUPR) were obtained; meanwhile, in comparison with the known predictive model with the same dataset, the KGDCMI achieved the best prediction accuracy. In addition, in three groups of comparative experiments, we verified an optimal classifier, the most suitable K value of *K-mer* for RNA fragment extraction, and feature extraction dimensions respectively. In the prediction results of the proposed method, seven of the 10 pairs with the highest predicted scores were confirmed in published literature, meaning that the method is powerful and feasible.

## Materials and Methods

### Dataset

Circbank (Liu, Wang, Shen, Yang, and Ding 2019) is a publicly available database including the five features of circRNAs, such as a novel naming system of circRNAs based on the circRNAs host genes and the conservation of circRNAs. The Circbank contains approximately 140,000 human circRNAs and 1917 human miRNAs. After removing redundant data, we obtained 9589 circRNA–miRNA interaction pairs, including 2115 circRNAs and 821 miRNAs from the Circbank database.

CircR2Cancer database (Lan et al., 2020) is a manually managed database containing 1439 circRNA–cancer associations between 1135 circRNAs and 82 cancers, and all these associations have been verified by experiments and existing literature. We obtained a total of 318 pairs of circRNA–miRNA between 238 circRNAs and 230 miRNAs from the circR2Cancer database.

After integrating these data from two databases, we finally obtained a total of high-quality 9905 circRNA–miRNA interaction pairs between 2346 circRNAs and 962 miRNAs.

### Extracting Sequence Features of RNAs by *K-mer* Algorithm

Counting all *K-mers* (substrings of length *K*) in RNA sequences is usually used as an important and common step in bioinformatics analysis, like variants detection, transcriptome assembly, and read error correction. Related studies have confirmed that RNA sequences contain abundant information. To obtain more comprehensive attribute information on RNAs, we used the *K-mer* algorithm to obtain a sparse matrix from the RNA sequences, which represent the RNA’s attribute features.

For a given circRNA or miRNA sequence, *K-mer* was used to split them into subsequences. It scanned each RNA sequence from beginning to the end with a k nucleotides window, one nucleic acid once a time. For a sequence of length *K*, we could obtain *4* ^*K*^ different possible *K-mers*. Different *K* values determine different lengths of vectors. For example, *3-mers* of circRNA can be represented as *AAA, AAC, …, TTG,* and *TTT*, and the number of possible *3-mers* is *l-3 + 1*, whereas *5-mers* can be represented as *AAAAA, AAAAC, …, TTTTG, TTTTT*, and the possible *5-mers* is *l-5 + 1*. After processing circRNA and miRNA sequences, we obtained the sequence characteristic representation matrices of each RNA, which can be represented as

$$K_{circRNA}=2346\times 4^{5}$$ (1)

$$K_{miRNA}=962\times 4^{2}$$ (2)

**FIGURE 1:**
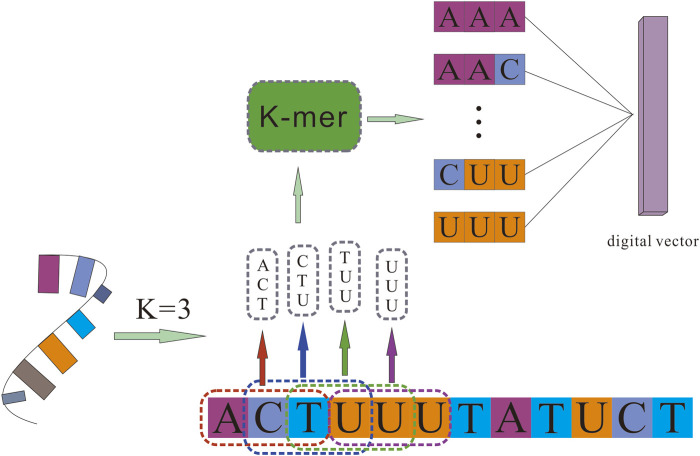
The *K-mer* algorithm for sequence feature extraction.

The detail of the *K-mer* algorithm is shown in Figure 1.

### Gaussian Interaction Profile Kernel Similarity for CircRNA and miRNA

Homologous RNAs with similar associations may have receptors with similar phenotypes; here, we increased the RNA Gaussian interaction profile kernel similarity to represent circRNA and miRNA similarity.

First, with the adjacent matrix, *P*_*c × m*_ represents the associations of circRNAs and miRNAs, and the number of circRNAs and miRNAs is defined as c and m. When circRNA *i* is related to miRNA *j*, the value of the matrix *P*_*(ij)*_ equals 1, and is otherwise 0. Each row of matrix,*P*_*c × m*_ represents a circRNA interaction profile, and the column represents a miRNA interaction profile. Furthermore, the gastric inhibitory polypeptide (GIP) kernel of circRNA can be calculated by the following formula:

$$G_{C}\left(c_{i},c_{j}\right)=\exp\left(-\lambda_{c}||LP\left(c_{i}\right)-LP\left(c_{j}\right){\|}^{2}\right)$$ (3)

where *G*_*C*_ (*c*_*i*_ *, c*_*j*_) denotes the GIP kernel similarity between circRNA *c*_*i*_, and circRNA *c*_*j*_, *λ*_*c*_ is an adjustable parameter, which controls the kernel bandwidth:

$$\lambda_{c}=\lambda_{c}/\left(\frac{1}{nc}\sum_{i=1}^{nc}||Lp\left(c_{i}\right)|{|}^{2}\right)$$ (4)

$\lambda_{c}$ is defined as one according to the previous study (Van Laarhoven et al., 2011).

Likewise, the GIP kernel similarity between miRNA $m_{i}$ and miRNA $m_{j}$ is calculated as

$$G_{M}\left(m_{i},m_{j}\right)=\exp(-\lambda_{m}||LP\left(m_{i}\right)-LP\left(m_{j}\right){\|}^{2}$$ (5)

$$\lambda_{m}=\lambda_{m}\prime /\left(\frac{1}{nm}\sum_{i=1}^{nm}||Lp\left(m_{i}\right)|{|}^{2}\right)$$ (6)

### Sparse Autoencoder to Extract Features

RNA sequences contain a great deal of valuable information, and we believe that using the *K-mer* algorithm can effectively transform sequence information into a digital vector containing rich attribute information. When using the *K-mer* algorithm, the value of *K* ranges from 2 to 5, which is the most effective empirical parameter verified. However, the length of RNA sequences is not uniform. When the value of *K* is too small, useful information will be lost. On the contrary, it will cause “noise” disturbance and increase the computational overhead.

Principal component analysis is a data dimensionality reduction and feature extraction method widely used before data are preprocessed by other algorithms. Furthermore, it removes noise and useless information about features to maximize the information value of features and improve the algorithm accuracy. In this work, we used the SAE (Ng 2011) with sparse penalty terms to obtain a more effective and comprehensive eigenvector.

SAE is a three-layer neural network including an input layer, an output layer, and a hidden layer that include two functions: encoding and decoding.

The function of encoding the layer is mapping the input feature x to the hidden layer *H*_*2*_

$$H_{2}=\sigma \left(W_{H_{1}}x\left(i\right)+b_{H_{1}}\right)$$ (7)

where *x* is the origin-dimensional input data vector, *W* is the connection parameter between the input and the hidden layer, and *b* represents a function offset.

Select *σ*(⋅) as the network’s activation function:

$$\sigma \left(x\right)=\frac{1}{\left(1+e^{-x}\right)}.$$ (8)

*H*_*2*_= (*h*_*1*_ *, h*_*2*_ *, …, h*_*l*_) is the vector output from the hidden layer.

The average activation amount of the activated hidden unit t is

$${\hat{\rho }}_{j}=\frac{1}{n}\sum_{i=1}^{n}\left[a_{j}\left(x\left(i\right)\right)\right]$$ (9)

*a*_*j*_(*x*) denotes the activation of hidden unit t.

SAE adds the sparsity penalty term to the target function, which constraints on the hidden layer to maintain low-average-activation values:

$$P=\sum_{j=1}^{h_{2}}KL\left(\rho ||{\hat{\rho }}_{j}\right)$$ (10)

*h*_*2*_ is the number of neurons in the hidden layer, and *P* reflects the degree of penalization $\hat{\rho }$ _*j*_ deviating from *ρ*.

The sparsity penalty term is expressed as the *Kullback–Leibler* (*KL*) divergence. In general, *ρ* is a sparsity parameter, which is a small value close to 0:

$$KL\left(\rho ||{\hat{\rho }}_{j}\right)=\rho \log\frac{\rho }{\hat{\rho }}+\left(1-\rho \right)\log\frac{1-\rho }{1-{\hat{\rho }}_{j}}$$ (11)

If $\hat{\rho }$ = *ρ*, *KL* (*ρ||* $\hat{\rho }$ _*j*_) = 0; Otherwise, *KL* increases monotonically as the gap grows.

The cost function of sparse penalty term is defined as

$$F_{C}\left(W,b\right)=F\left(W,b\right)+\kappa \sum KL\left(\rho ||\hat{\rho }\right),$$ (12)

where *F* (*W, b*) is the cost function of the neural networks, and *κ* is the weight of the sparse penalty.

### High-Order Proximity Reserved Embedding

Graph-embedding algorithm is an effective method to mine for hidden information that can map graphs into the vector space and retain the structure and inherent attributes of graphs. In this part, we constructed a bipartite graph of the circRNA–miRNA interactions and used a graph-embedding algorithm, HOPE (Ou et al., 2016) to capture hidden information between nodes in a large-scale network.

For a given graph *Gn =<V, E>*, *V* is a collection of vertices, and *E* represents the directed edge sets. *HOPE* embeds *Gn* into a vector space, where the asymmetric transitivity and the structure of the graph are preserved. HOPE preserves the asymmetric transitivity by approximating high-order proximity, and it adopts the *L2-norm* below as the minimized loss function:

$$\min||M-N^{s}\cdot N^{t⊤}|{|}_{F}^{2}$$ (13)

*M* is a high-order proximity matrix, where *M*_*ij*_ is the proximity between *v*_*i*_ and *v*_*j*_, and *N* = [*N* ^*s*^ *⋅ N* ^*t*^] represents the embedding matrix, *N* ^*s*^, *N* ^*t*^ ϵ γ^α × β^are the source embedding vectors and target embedding vectors respectively, where *β* is the dimension of the embeddings. Many high-order approximations reflect asymmetric transfer properties, *HOPE* adopts a general formulation to facilitate the approximation of these proximities:

$$M=M_{g}^{-1}\cdot M_{l}$$ (14)

where *M*_*g*_, and *M*_*l*_ are polynomial matrices. Furthermore, *HOPE* uses singular value decomposition (SVD) to obtain an optimal *rank-K* approximation of the proximity matrix *M* and corresponding singular vectors to construct embedding vectors:

$$M=\sum_{i=1}^{N}\sigma_{i}v_{i}^{s}v_{i}^{tT}$$ (15)

where *σ*_*i*_ is a singular value sorted in descending order, and $v_{i}^{s}$ $v_{i}^{t}$ are corresponding singular vectors of *σ*_*i*_. The obtained optimal embedding vectors are

$$N^{s}=\left[\sqrt{\sigma_{1}}\cdot v_{1}^{s},\cdots ,\sqrt{\sigma_{k}}\cdot v_{k}^{s}\right]$$ (16)

$$N^{t}=\left[\sqrt{\sigma_{1}}\cdot v_{1}^{t},\cdots ,\sqrt{\sigma_{k}}\cdot v_{k}^{t}\right]$$ (17)

### Deep Neural Network

In recent years, as a new machine learning technology, the neural network has become a widely used method in many fields and achieved satisfactory results. We used the DNN to effectively learn and fuse multidimensional features and predict potential circRNA–miRNA interactions.

After SAE extracted the attribute feature and HOPE processed the embedded feature, the extracted attribute feature and behavior feature were concatenated and formed a complete sample feature vector. Then, the complete feature vector was fed into the DNN composed of an input layer, multiple hidden layers, and an output layer to obtain the final prediction of the interaction between each circRNA and miRNA.

DNN was fed features from the input layer, and then the hidden layers transformed data in a non-linear way. At last, the learned features were calculated to the output layer. In the whole DNN model, the neuron units in layer n were connected to the layer (n − 1), and the output data were generated by non-linear transformation function *f* (⋅):

$$D_{i+1}=f\left(\sum_{i=1}^{H}w_{i}d_{i}+b_{i}\right)$$ (18)

where *H* represents the number of hidden neurons, and *w*_*i*_ and *b*_*i*_ are the weight and bias of neurons respectively. In our method, the rectified linear unit was used to capture hidden patterns within the feature and reduce gradient vanishing:

$$F_{RELU}\left(x\right)=\max\left(0,x\right)$$ (19)

and *sigmoid* was utilized to project the predicted value within a reasonable range:

$$F_{sigmoid}\left(x\right)=\frac{1}{1+e^{-x}}$$ (20)

**FIGURE 2:**
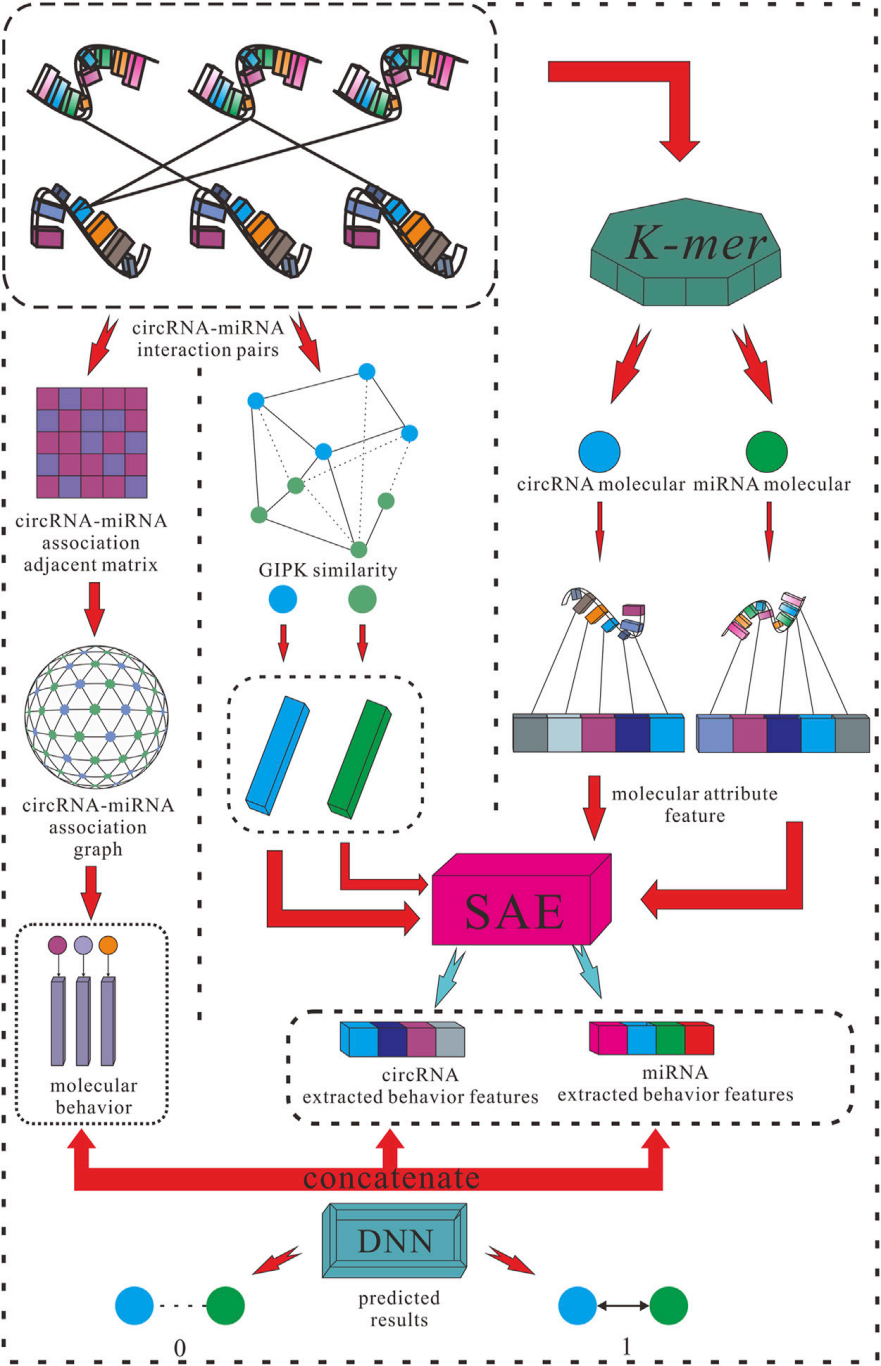
Flowchart of KGDCMI.

We selected *binary_crossentropy* as the cost function to minimize the objective function to minimize the loss, which is shown as follows:

$$F_{\cos t}=-\frac{1}{L}\sum_{j}^{L}y_{j}\cdot \log{\hat{y}}_{j}+\left(1-y_{j}\right)\cdot \log\left(1-{\hat{y}}_{j}\right)$$ (21)

where *L* is the length of the output size, *y*_*j*_ represents the true label for samples 0 or 1, respectively, and $\hat{y}$ _*j*_ is the predicted probability of the point being positive. For a given positive sample *y* = 1, the closer the predicted value is to 1, the smaller the loss value is, and vice versa. In an ideal sense, we hope the loss value approaches 0 infinitely. The specific step is shown in Figure 2.

## Results

### Evaluation Criteria

In this study, the samples were first divided into a training, validation, and test set. Taking 70% of samples as the training set to construct the representation vector and train the DNN classifier, 20% of samples were regarded as a validation set, and the remaining 10% of them were treated as a test set to evaluate the performance of the model. At last, the mean and standard deviation of the results of five experiments were calculated. Meanwhile, to fully and fairly demonstrate the performance of our method, we introduced five evaluation criteria to comprehensively evaluate the results of the model, which assesses the robustness and effectiveness of the model. The evaluation criteria used include the *ACC.* (accuracy), *Sen.* (sensitivity), *Spec.* (specificity), *Prec.* (precision), and *MCC.* (Matthews correlation coefficient) to ensure comprehensiveness and fairness of the results. The calculation formula is as follows:

$$Acc.=\frac{\left(TN+TP\right)}{\left(TN+TP+FN+FP\right)}$$ (22)

$$Sen.=\frac{TP}{\left(TP+FN\right)}$$ (23)

$$Spec.=\frac{TN}{\left(TN+FP\right)}$$ (24)

$$\mathrm{Pr}ec.=\frac{\left(TP\right)}{\left(TP+FP\right)}$$ (25)

$$Mcc.=\frac{\left(TP\times TN-FP\times FN\right)}{\sqrt{\left(\left(TP+FP\right)\times \left(TP+FN\right)\times \left(TN+FP\right)\times \left(TN+FN\right)\right)}}$$ (26)

where *TP* (true positive) is the count of true interactions predicted to have interacting circRNA–miRNA samples; *TN* (true negative) is the number of true interactions predicted to have non-interacting circRNA–miRNA samples; *FN* (false negative) is the count of interacting circRNA–miRNA samples that are predicted to have no interaction; and *FP* (false positives) refers to the number of non-interacting circRNA–miRNA samples predicted to interact.

### Assessment of Prediction Ability

In this part, to verify the practicability and performance of the proposed method, the dataset, including data based on the Circbank database and circR2Cancer database, has been used to evaluate the method. Tables 1 list the experimental results of the proposed method, the results on the dataset were as follows: the average *ACC.* of experiments is 82.65%, *Sen.* is 80.19%, *Spec.* is 85.10%, *Prec.* is 84.35%, *MCC.* is 65.38%, AUC is 89.30%, and AUPR is 87.67%. The standard deviations of these evaluation criteria are 0.41, 1.02, 1.44, 1.16, 0.87, and 0.28 respectively. Among the five-experiment validation, the highest AUC was 89.63%, and the lowest was 89.04%.

**TABLE 1:** Five-fold cross-validation results performed by KGDCMI.

Test Set	ACC. (%)	Sen. (%)	Spec. (%)	Prec. (%)	MCC (%)
1	82.00	80.62	83.39	82.92	64.03
2	82.94	78.55	87.33	86.11	66.13
3	82.74	80.11	85.36	84.55	65.56
4	83.04	81.32	84.76	84.21	66.12
5	82.51	80.36	84.65	83.97	65.08
Average	82.65±0.41	80.19±1.02	85.10±1.44	84.35±1.16	65.38±0.87

**FIGURE 3:**
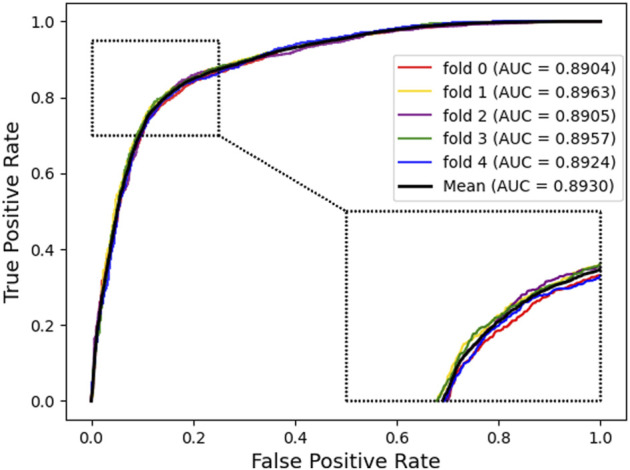
Receiver operating characteristic curves generated by KGDCMI.

**FIGURE 4:**
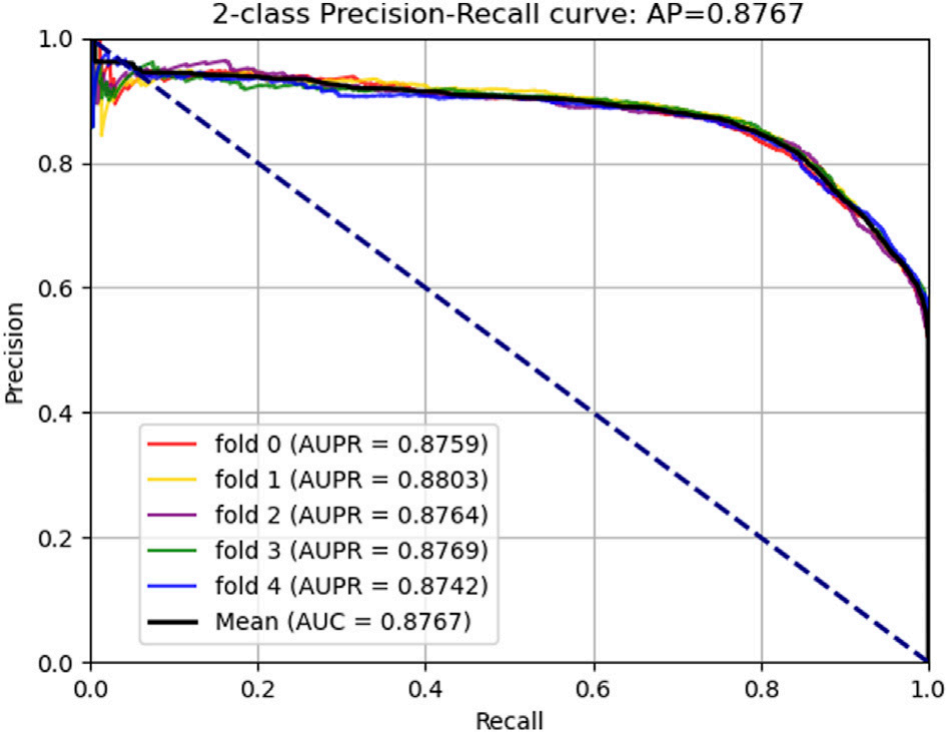
Area under the precision–recall curves generated by KGDCMI.

In addition, we plotted the receiver operating characteristic curve (ROC) and precision–recall (PR) for visualization, while we calculated the area under ROC (AUC) and area under PR (AUPR) separately to facilitate comparison with other methods. The AUC and PR curves are shown in Figures 3, 4, respectively.

### Comparison With Related Models

To demonstrate the superiority of KGDCMI in the prediction of the circRNA–miRNA interaction, we compared the performance of the proposed method with the only existing model in this field. Since few models use computational methods to predict the circRNA–miRNA interactions, we also compared our method with several models in other highly related fields. In our comparative experiment, all models used 9589 pairs of data based on the Circbank and adopted five-fold cross-validation. At last, it was found that our method achieved the best results.

To the best of our knowledge, the CMIVGSD (Qian et al., 2021) is the only known computational framework for predicting the circRNA–miRNA interactions, which obtains the linear features by SVD and uses the graph variational autoencoder to generate the non-linear features. At last, the LightGBM classifier is used to predict the circRNA–miRNA interactions. The deep matrix factorization prediction model (Liu et al., 2020) uses a neural network with an embedding layer to obtain a low-dimensional dense vector and realize matrix factorization, which is applied to obtain final scores of microbe–disease pairs. The NTSHMDA (Luo et al., 2018) constructs a heterogeneous network by similarity network and uses a method based on the random walk to predict the microbe–disease association. Furthermore, the AE–RF (Deepthi et al., 2021) integrates a similar network to construct features, uses a deep autoencoder to extract hidden features, and trains the random forest classifier to predict the association between circRNA and disease. The Deep Matrix Factorization CircRNA-Disease Association (Lu et al., 2020) uses a projection layer to learn latent features and applies a multi-layer neural network to predict the association between circRNA and disease.

Table 2 shows the AUC and AUPR of our model and the other five models under the five-fold cross-validation. Our model obtains the best AUC and AUPR among the five models. Moreover, our model achieves the highest accuracy in the field of circRNA–miRNA interaction prediction, which is 2.37% higher than the second-best model.

**TABLE 2:** Performance comparison of five methods based on five-fold cross-validation.

Methods	DMFCDA	AE–RF	DMFMDA	NTSHMDA	CMIVGSD	KGDCMI
AUC	0.7321	0.7662	0.7922	0.8526	0.8804	0.9041
AUPR	0.7115	0.8239	0.8230	0.8772	0.8629	0.8937

### Comparison With Traditional Classifier

**FIGURE 5:**
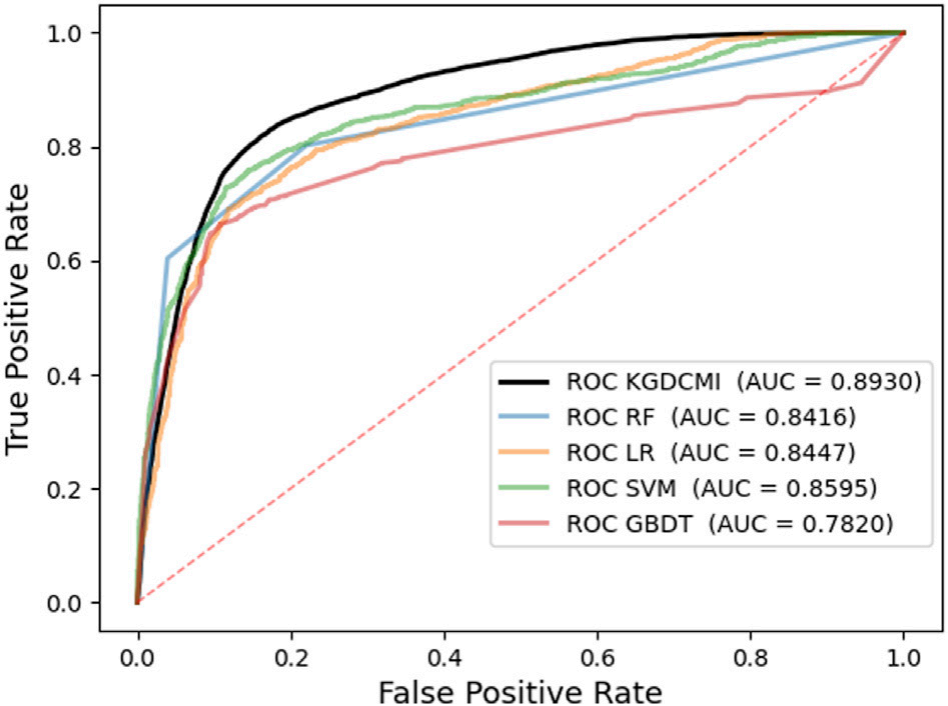
Performance comparison of five traditional classifiers and DNN in terms of prediction.

In the prediction method of the KGDCMI, we applied the DNN to fuse multidimensional features and classify the interaction between circRNA and miRNA. To reflect the superiority of our classification strategy, we used some traditional classifiers to replace our classification method and compare the results. In a more concrete sense, we kept the multiple features of the KGDCMI unchanged and conducted independent experiments with four different classifiers, including the random forest classifier (Breiman 2001), logistic regression classifier (LaValley 2008), support vector machine classifier (Cortes and Vapnik 1995), and gradient boosting decision tree (Friedman 2001) to replace DNN for prediction, the prediction results are shown in Figure 5.

It can be seen in Figure 5 that the results of the DNN classifier are significantly better than the five traditional classifiers. The results of these experiments indicated that DNN is applicable to the proposed method. The main reason for this is that the DNN has a better fusion for multidimensional features, which can give full play to the advantages of behavior characteristics and attribute characteristics in our method. Therefore, the DNN shows a better performance than the traditional classifiers when using the same feature description.

### Parameter Settings for Attribute Feature Dimension

This study used the *K-mer* method to extract attribute features from RNA sequences. For a given RNA, we can obtain *4* ^*k*^-dimensional vectors, which depend on the size of the value *K*. The value sizes {2,3,4,5} of *K* are frequently used values.

However, miRNA is a non-coding RNA transcript with an average length of 21 nucleotides (Buermans et al., 2010), and circRNA is a long-stranded RNA molecule, where over 14% of circRNAs in humans and over 10% of circRNA in mouse are more than 800 nucleotides in length (Wu et al., 2020). Due to the great difference in the sequence length between miRNA and circRNA, we need to adopt different *K* values for different RNAs to ensure that the most valuable features can be extracted.

In this part, we treated miRNA and circRNA with *K* ∈ {2, 3, 4, 5} respectively and compared the experimental results produced by different *K* values to obtain the most appropriate *K* value for each RNA.

Table 3 shows the performance of our model under different K values. To independently reflect the impact of different dimensions on the performance of the model, in the comparative experiment of K value, we do not add similarity descriptors and do not use SAE to extract features.

**TABLE 3:** Performances of different *K* values.

*K-value*	Sen. (%)	Spec. (%)	Prec. (%)	Acc. (%)	AUC. (%)
*K*_*miRNA*_ = 2, *K*_*circRNA*_ = 2	74.89	86.46	84.70	80.67	86.25
*K*_*miRNA*_ = 2, *K*_*circRNA*_ = 3	77.20	85.29	84.00	81.25	86.77
*K*_*miRNA*_ = 2, *K*_*circRNA*_ = 4	75.49	86.55	84.88	81.02	86.33
*K*_*miRNA*_ = 2, *K*_*circRNA*_ = 5	76.77	86.15	84.72	81.46	87.21
*K*_*miRNA*_ = 3, *K*_*circRNA*_ = 2	76.75	85.17	83.83	80.96	86.39
*K*_*miRNA*_ = 3, *K*_*circRNA*_ = 3	77.27	85.29	84.04	81.27	86.63
*K*_*miRNA*_ = 3, *K*_*circRNA*_ = 4	75.72	86.90	85.26	81.31	86.43
*K*_*miRNA*_ = 3, *K*_*circRNA*_ = 5	77.52	85.56	84.31	81.54	86.97
*K*_*miRNA*_ = 4, *K*_*circRNA*_ = 2	77.79	84.86	83.91	80.83	86.16
*K*_*miRNA*_ = 4, *K*_*circRNA*_ = 3	78.44	85.04	84.00	80.73	86.17
*K*_*miRNA*_ = 4, *K*_*circRNA*_ = 4	77.08	85.57	84.33	80.33	86.43
*K*_*miRNA*_ = 4, *K*_*circRNA*_ = 5	77.67	84.42	84.21	80.74	86.61
*K*_*miRNA*_ = 5, *K*_*circRNA*_ = 2	76.70	84.50	83.73	79.10	85.26
*K*_*miRNA*_ = 5, *K*_*circRNA*_ = 3	76.58	84.93	83.68	80.25	85.89
*K*_*miRNA*_ = 5, *K*_*circRNA*_ = 4	76.74	85.00	83.93	79.11	85.75
*K*_*miRNA*_ = 5, *K*_*circRNA*_ = 5	76.87	85.52	83.66	79.70	85.92

**FIGURE 6:**
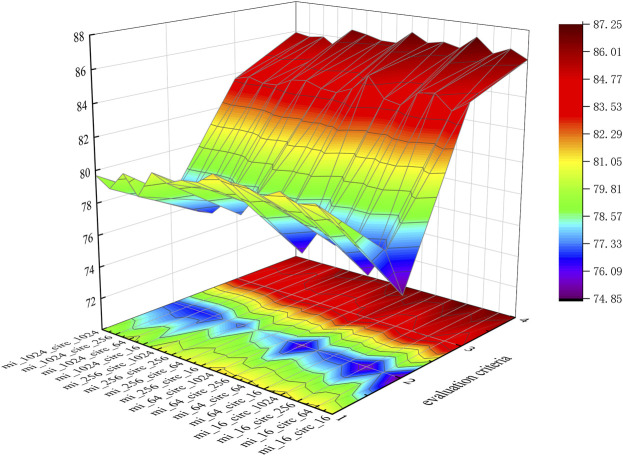
Performances of different *K* values.

To intuitively represent each group of data, we visualize the data in three-dimensional space, as shown in Figure 6.

In Figure 6, evaluation criteria 1 through 4 represent Sen., Spec., Prec., and AUC., respectively. Meanwhile, mi_16 indicates that the dimensions of miRNA are 16, which means *K*_*miRNA*_ = 2, which is the same as for circRNA.

According to Table 3 and Figure 6, when mi_16_circ_1024 (*K*_*miRNA*_ = 2, *K*_*circRNA*_ = 5), KGDCMI obtained the highest accurate value.When mi_64_circ_1024 (*K*_*miRNA*_ = 3, *K*_*circRNA*_ = 5) and mi_16_circ_64 (*K*_*miRNA*_ = 2, *K*_*circRNA*_ = 3), the method achieved the second and third highest results, respectively.

The *K* value of the *K-mer* algorithm represents the abundance of divided sequence fragments, larger *K* values tend to produce more representative features. However, for miRNA with a short sequence, when *K > 3*, the characteristic matrix generated by *K-mer* becomes sparser, and the performance of KGDCMI begins to decline. For circRNA with a long sequence, when 3<K≤5 , the method achieves satisfactory results, which shows that the algorithm can effectively extract features, but not sufficiently. When *K* = 5, we obtain the best performance.

According to our experiment, when *K*_*miRNA*_ = 2 and *K*_*circRMA*_ *= 5*, attribute feature extraction based on *K-mer* can obtain the best effect. This is the first analysis of the different dimensions of feature extraction from RNA sequence length in the field of circRNA–miRNA interaction prediction, and we believe this research will provide a reference for future experiments.

### Different Dimensions Based on Sparse Autoencoder

**FIGURE 7:**
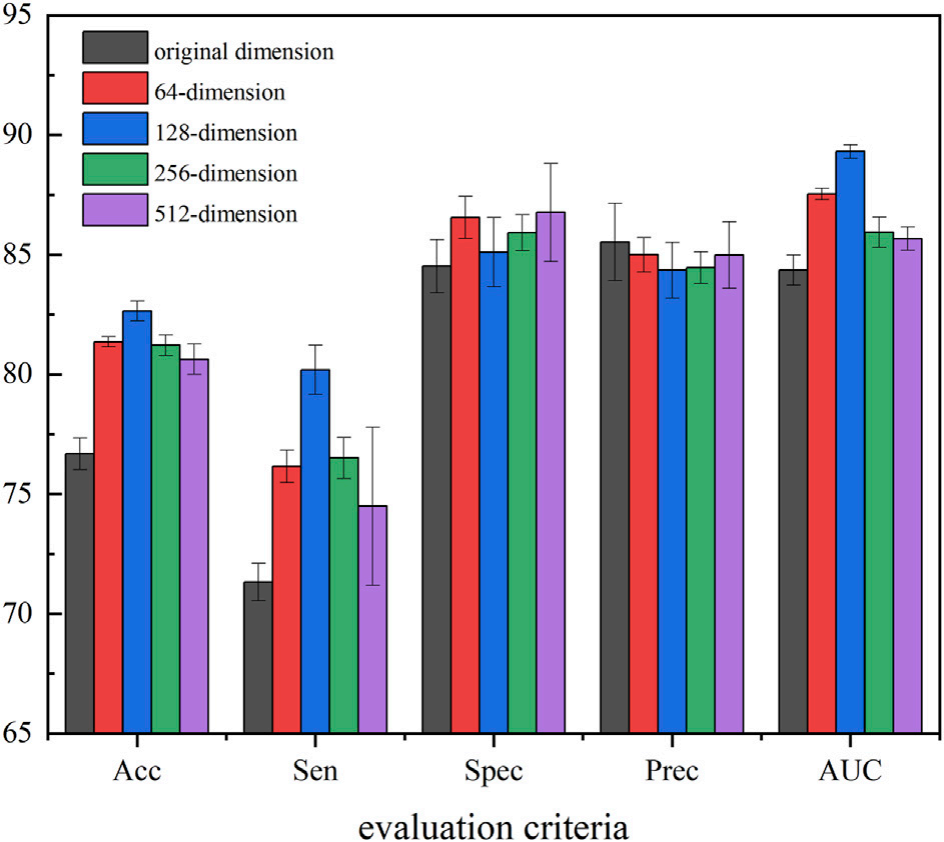
Performance of five-dimensional compression to extract features.

In our method, to obtain the most representative feature descriptor, SAE was employed to process the resulting eigenvectors to learn the hidden and relatively sparse features. To further evaluate the efficiency of the proposed feature extraction strategy and determine the best extraction dimension, we applied SAE to extract features with different dimensions as the final behavior feature. In detail, we selected *5-mers* circRNA and *2-mers* miRNA for which *K* showed the best performance in the previous section, and used 64-, 128-, 256-, and 512-dimension final behavior features extracted by SAE to perform experiments. Figure 7 provides experiment results of the different feature dimensions of the proposed method.

Figure 7 shows that compression of all four dimensions improves the accuracy of the model, which suggests that the use of SAE can effectively improve robustness, generalization ability, and accuracy. In all five evaluation criteria, 128-dimension compression achieves the best results in three evaluation criteria, Therefore, in this experiment, we set the SAE compression dimensions at 128.

## Case Studies

To verify the performance of our model in the real environment, we implemented the case study based on our dataset. First, we used known circRNA–miRNA interactions to construct the features vectors and train the model. Second, the trained model was used to predict the unknown association interactions, and finally, we obtained the final score of prediction of each pair after sorting the results from large to small, the top 10 results are shown in Table 4. We can see from table 4 that 7 of the top 10 circRNA–miRNA interactions were confirmed in PubMed. The remaining three unconfirmed pairs of interaction were not verified by biological experiments, but there is no doubt that interaction is possible between them.

**TABLE 4:** The top 10 results predicted in our model based on the dataset.

Num	CircRNA	miRNA	Evidence
1	hsa_circ_0006916	hsa-miR-522-3p	PMID:29726904
2	hsa_circ_0002142	hsa-miR-625-5p	PMID:30988674
3	hsa_circ_0000977	hsa-miR-874-3p	PMID:29454093
4	hsa_circ_0041089	hsa-miR-3192-5p	unconfirmed
5	hsa_circ_0041103	hsa-miR-103a-3p	PMID:27484176
6	hsa_circ_0007915	hsa-miR-106a-3p	PMID:28727484
7	hsa_circ_0000673	hsa-miR-767-3p	unconfirmed
8	hsa_circ_100242	hsa-miR-145-5p	PMID:32218853
9	hsa_circ_0092306	hsa-miR-197-3p	PMID:31689616
10	hsa_circ_0089776	hsa-miR-6752-5p	unconfirmed

## Conclusion

Due to the high labor cost and the production time of biological methods, computational methods have increasingly received attention and have been used to predict the association of different molecules. Predicting the interaction between circRNA and miRNA can not only save resources and time but also help to find the potential relationship between molecules and facilitate the understanding of complex disease mechanisms (Lei et al., 2021). At present, there is only one computational prediction model for circRNA and miRNA interaction, to the best of our knowledge. This study proposed a method called KGDCMI to predict the interaction between the circRNA and miRNA.

First, we used the *K-mer* algorithm and Gaussian kernel similarity to obtain the digital descriptor representing the attribute characteristics of RNA. Second, SAE was used to remove the redundancy and noise of attribute features to obtain the final attribute vectors. Next, we used HOPE to capture behavior information in molecular association networks. At last, we used a DNN for feature fusion and get the final predict score.

With the same dataset, our model obtained the highest AUC (90.41%) and AUPR (89.37%), which is 2.37% and 3.08% higher than the second-best model, respectively. Meanwhile, we compared and discussed the extraction of fragments with different lengths of molecular sequences for the first time, and we obtained the most suitable *K* value for miRNA and circRNA, which we believe will facilitate future research. The results predicted by the model were verified in case studies. There is no doubt that our model is an effective computational tool to predict the circRNA–miRNA interaction.

## Data Availability

The datasets for this paper can be found in the Circbank http://www.circbank.cn/, CircR2Cancer http://www.biobdlab.cn:8000/. The data and source code can be found at https://github.com/1axin/KGDCMI. KGDCMI is also publicly available as an online predictor at http://120.77.11.78/KGDCMI/.

## References

[B1] Armakola M. Higgins M. J. Figley M. D. Barmada S. J. Scarborough E. A. Diaz Z. (2012). Inhibition of RNA Lariat Debranching Enzyme Suppresses TDP-43 Toxicity in ALS Disease Models. Nat. Genet. 44, 1302–1309. 10.1038/ng.2434 23104007 PMC3510335

[B2] Breiman L. 2001. Random Forests.45:5–32. 10.1023/a:1010933404324

[B3] Buermans H. P. Ariyurek Y. van Ommen G. den Dunnen J. T. 't Hoen P. A. (2010). New Methods for Next Generation Sequencing Based microRNA Expression Profiling. BMC Genomics 11, 716–16. 10.1186/1471-2164-11-716 21171994 PMC3022920

[B4] Chen L. Shan G. (2021). CircRNA in Cancer: Fundamental Mechanism and Clinical Potential. Cancer Lett. 505, 49–57. 10.1016/j.canlet.2021.02.004 33609610

[B5] Chen P. Nie Z-Y. Liu X-F. Zhou M. Liu X-X. Wang B. J. N. (2022). CircXRCC5, as a Potential Novel Biomarker, Promotes Glioma Progression via the miR-490-3p/XRCC5/CLC3 ceRNA Network. 10.1016/j.neuroscience.2021.12.037 35436516

[B6] Cocquerelle C. Daubersies P. Majérus M. A. Kerckaert J. P. Bailleul B. (1992). Splicing with Inverted Order of Exons Occurs Proximal to Large Introns. EMBO J. 11, 1095–1098. 10.1002/j.1460-2075.1992.tb05148.x 1339341 PMC556550

[B7] Cortes C. Vapnik V. (1995). Support-vector Networks. Mach. Learn 20, 273–297. 10.1007/bf00994018

[B8] Deepthi K. Jereesh A. S. Therapy (2021). Inferring Potential CircRNA-Disease Associations via Deep Autoencoder-Based Classification. Mol. Diagn Ther. 25, 87–97. 10.1007/s40291-020-00499-y 33156515

[B9] Fan C. Lei X. Fang Z. Jiang Q. Wu F-X. J. D. (20182018). CircR2Disease: A Manually Curated Database for Experimentally Supported Circular RNAs Associated with Various Diseases. 10.1093/database/bay044 PMC5941138 29741596

[B10] Friedman J. (2001). Greedy Function Approximation: A Gradient Boosting Machine, 1189–1232.

[B11] Glažar P. Papavasileiou P. Rajewsky N. J. R. (2014). circBase a database circular RNAs 20, 1666–1670. 10.1261/rna.043687.113 PMC4201819 25234927

[B12] Grishok A. Pasquinelli A. E. Conte D. Li N. Parrish S. Ha I. (2001). Genes and Mechanisms Related to RNA Interference Regulate Expression of the Small Temporal RNAs that Control *C. elegans* Developmental Timing. Cell. 106, 23–34. 10.1016/s0092-8674(01)00431-7 11461699

[B13] Hansen T. B. Jensen T. I. Clausen B. H. Bramsen J. B. Finsen B. Damgaard C. K. (2013). Natural RNA Circles Function as Efficient microRNA Sponges. Nature 495, 384–388. 10.1038/nature11993 23446346

[B14] Hayes J. Peruzzi P. P. Lawler S. (2014). MicroRNAs in Cancer: Biomarkers, Functions and Therapy. Trends Mol. Med. 20, 460–469. 10.1016/j.molmed.2014.06.005 25027972

[B15] Hsu M.-T. Coca-Prados M. (1979). Electron Microscopic Evidence for the Circular Form of RNA in the Cytoplasm of Eukaryotic Cells. Nature 280, 339–340. 10.1038/280339a0 460409

[B16] Kulcheski F. R. Christoff A. P. Margis R. (2016). Circular RNAs Are miRNA Sponges and Can Be Used as a New Class of Biomarker. J. Biotechnol. 238, 42–51. 10.1016/j.jbiotec.2016.09.011 27671698

[B17] Lan W. Zhu M. Chen Q. Chen B. Liu J. Li M. (2020). CircR2Cancer: A Manually Curated Database of Associations between circRNAs and cancers.2020. 10.1093/database/baaa085 PMC7661096 33181824

[B18] LaValley M. P. (2008). Logistic Regression. Circulation 117, 2395–2399. 10.1161/circulationaha.106.682658 18458181

[B19] Lei X. Mudiyanselage T. B. Zhang Y. Bian C. Lan W. Yu N. (2021). A Comprehensive Survey on Computational Methods of Non-coding RNA and Disease Association Prediction, 22, bbaa350. 10.1093/bib/bbaa350 33341893

[B20] Li L. Gao Z. Wang Y.-T. Zhang M.-W. Ni J.-C. Zheng C.-H. (2021). SCMFMDA: Predicting microRNA-Disease Associations Based on Similarity Constrained Matrix Factorization. PLoS Comput. Biol. 17, e1009165. 10.1371/journal.pcbi.1009165 34252084 PMC8345837

[B21] Li Z. Huang C. Bao C. Chen L. Lin M. Wang X. (2015). Exon-intron Circular RNAs Regulate Transcription in the Nucleus. Nat. Struct. Mol. Biol. 22, 256–264. 10.1038/nsmb.2959 25664725

[B22] Liu M. Wang Q. Shen J. Yang B. B. Ding X. (2019). Circbank: a Comprehensive Database for circRNA with Standard Nomenclature. RNA Biol. 16, 899–905. 10.1080/15476286.2019.1600395 31023147 PMC6546381

[B23] Liu Y. Wang S. Zhang J. Zhang W. Zhou S. Li W. (2020). Dmfmda: Prediction of Microbe-Disease Associations Based on Deep Matrix Factorization Using Bayesian Personalized Ranking. IEEE/ACM Trans. Comput. Biol. Bioinform PP, 1763–1772. 10.1109/TCBB.2020.3018138 32816678

[B24] Lu C. Zeng M. Zhang F. Wu F. Li M. Wang J. (2020). Deep Matrix Factorization Improves Prediction of Human circRNA-Disease Associations. IEEE J. Biomed. Health Inf. PP, 891–899. 10.1109/JBHI.2020.2999638 32750925

[B25] Luo J. Long Y. bioinformatics (2020). NTSHMDA: Prediction of Human Microbe-Disease Association Based on Random Walk by Integrating Network Topological Similarity. IEEE/ACM Trans. Comput. Biol. Bioinform 17, 1341–1351. 10.1109/TCBB.2018.2883041 30489271

[B26] Memczak S. Jens M. Elefsinioti A. Torti F. Krueger J. Rybak A. (2013). Circular RNAs Are a Large Class of Animal RNAs with Regulatory Potency. Nature 495, 333–338. 10.1038/nature11928 23446348

[B27] Ng W. Ng W. (2011). Wedding Dress. Sparse autoencoder 72, 1–19. 10.4016/31240.01

[B28] Nigro J. M. Cho K. R. Fearon E. R. Kern S. E. Ruppert J. M. Oliner J. D. (1991). Scrambled Exons. Cell. 64, 607–613. 10.1016/0092-8674(91)90244-s 1991322

[B29] Ou M. Cui P. Pei J. Zhang Z. Zhu W. (2016). Proceedings of the Proceedings of the 22nd ACM SIGKDD International Conference on Knowledge Discovery and Data Mining. 10.1145/2939672.2939751 Asymmetric Transitivity Preserving Graph Embedding

[B30] Pan J. You Z-H. Li L-P. Huang W-Z. Guo J-X. Yu C-Q. (2022). DWPPI: A Deep Learning Approach for Predicting Protein–Protein Interactions in Plants Based on Multi-Source Information with a Large-Scale Biological Network, 10. 10.3389/fbioe.2022.807522 PMC8978800 35387292

[B31] Qian Y. Zheng J. Zhang Z. Jiang Y. Zhang J. Deng L. (2021). “CMIVGSD: circRNA-miRNA Interaction Prediction Based on Variational Graph Auto-Encoder and Singular Value Decomposition,” in Proceedings of the 2021 IEEE International Conference on Bioinformatics and Biomedicine (IEEE). 10.1109/bibm52615.2021.9669875

[B32] Qu S. Yang X. Li X. Wang J. Gao Y. Shang R. (2015). Circular RNA: a New Star of Noncoding RNAs. Cancer Lett. 365, 141–148. 10.1016/j.canlet.2015.06.003 26052092

[B33] Ren Z-H. Yu C-Q. Li L-P. You Z-H. Guan Y-J. Wang X-F. (2022). BioDKG–DDI: Predicting Drug–Drug Interactions Based on Drug Knowledge Graph Fusing Biochemical Information. 10.1093/bfgp/elac004 35368060

[B34] Sanger H. L. Klotz G. Riesner D. Gross H. J. Kleinschmidt A. K. (1976). Viroids Are Single-Stranded Covalently Closed Circular RNA Molecules Existing as Highly Base-Paired Rod-like Structures. Proc. Natl. Acad. Sci. U.S.A. 73, 3852–3856. 10.1073/pnas.73.11.3852 1069269 PMC431239

[B35] Siomi H. Siomi M. C. (2010). Posttranscriptional Regulation of microRNA Biogenesis in Animals. Mol. Cell. 38, 323–332. 10.1016/j.molcel.2010.03.013 20471939

[B36] Tao D. Liu Z. Wang L. Li C. Zhang R. Ni N. (2022). CircPAG1 Interacts with miR-211-5p to Promote the E2F3 Expression and Inhibit the High Glucose-Induced Cell Apoptosis and Oxidative Stress in Diabetic Cataract. Cell. Cycle 21, 708–719. 10.1080/15384101.2021.2018213 35174780 PMC8973334

[B37] Van Laarhoven T. Nabuurs S. B. Marchiori E. 2011. Gaussian Interaction Profile Kernels for Predicting Drug-Target Interaction.27:3036–3043. 10.1093/bioinformatics/btr500 21893517

[B38] Wu W. Ji P. Zhao F. (2020). CircAtlas: an Integrated Resource of One Million Highly Accurate Circular RNAs from 1070 Vertebrate Transcriptomes. Genome Biol. 21, 101–114. 10.1186/s13059-020-02018-y 32345360 PMC7187532

[B39] Xu H. Guo S. Li W. Yu P. (2015). The Circular RNA Cdr1as, via miR-7 and its Targets, Regulates Insulin Transcription and Secretion in Islet Cells. Sci. Rep. 5, 12453–12512. 10.1038/srep12453 26211738 PMC4515639

[B40] Yang J. Qi F. Tan B. Dai G. Chen R. Wan W. (202215391). circSPECC1 Promotes Bladder Cancer Progression via Regulating miR-136-5p/GNAS axis. 10.1016/j.prp.2022.153914 35523104

[B41] Yin M-M. Liu J-X. Gao Y-L. Kong X-Z. Zheng C-H. (2020). NCPLP: A Novel Approach for Predicting Microbe-Associated Diseases with Network Consistency Projection and Label Propagation. 10.1109/TCYB.2020.3026652 33119529

[B42] Zhang Z. Yang T. Xiao J. (2018). Circular RNAs: Promising Biomarkers for Human Diseases. EBioMedicine 34, 267–274. 10.1016/j.ebiom.2018.07.036 30078734 PMC6116471

[B43] Zhao Z. Wang K. Wu F. Wang W. Zhang K. Hu H. (2018). circRNA Disease: a Manually Curated Database of Experimentally Supported circRNA-Disease Associations. Cell. Death Dis. 9, 475–482. 10.1038/s41419-018-0503-3 29700306 PMC5919922

[B44] Zhou F. Yin M-M. Jiao C-N. Zhao J-X. Zheng C-H. Liu J-X. J. I. ToN. N. (2021). Predicting miRNA-Disease Associations through Deep Autoencoder with Multiple Kernel Learning. 10.1109/TNNLS.2021.3129772 34860656

